# Domestic Cook Book

**Published:** 1888-08

**Authors:** 


**Domestic Cook Book:** A Companion to Pulte's Domestic Physician. By Mrs. J. H. Pulte. George W. Smith. Cincinnati, 1888.

